# The Effect of a Subcutaneous Infusion of GLP-1, OXM, and PYY on Energy Intake and Expenditure in Obese Volunteers

**DOI:** 10.1210/jc.2017-00469

**Published:** 2017-04-04

**Authors:** Tricia Tan, Preeshila Behary, George Tharakan, James Minnion, Werd Al-Najim, Nicolai J. Wewer Albrechtsen, Jens J. Holst, Stephen R. Bloom

**Affiliations:** 1 Division of Diabetes, Endocrinology, and Metabolism, Hammersmith Hospital, Imperial College London, London W12 0HS, United Kingdom; 2 Department of Biochemical Sciences and Novo Nordisk Foundation Center for Basic Metabolic Research, Faculty of Health and Medical Sciences, University of Copenhagen, DK-2200 Copenhagen, Denmark

## Abstract

**Background:**

Roux-en-Y gastric bypass (RYGB) surgery is currently the most effective treatment of obesity, although limited by availability and operative risk. The gut hormones Glucagon-like peptide-1 (GLP-1), Peptide YY (PYY), and Oxyntomodulin (OXM) are elevated postprandially after RYGB, which has been postulated to contribute to its metabolic benefits.

**Objective:**

We hypothesized that infusion of the three gut hormones to achieve levels similar to those encountered postprandially in RYGB patients might be effective in suppressing appetite. The aim of this study was to investigate the effect of a continuous infusion of GLP-1, OXM, and PYY (GOP) on energy intake and expenditure in obese volunteers.

**Methods:**

Obese volunteers were randomized to receive an infusion of GOP or placebo in a single-blinded, randomized, placebo-controlled crossover study for 10.5 hours a day. This was delivered subcutaneously using a pump device, allowing volunteers to remain ambulatory. *Ad libitum* food intake studies were performed during the infusion, and energy expenditure was measured using a ventilated hood calorimeter.

**Results:**

Postprandial levels of GLP-1, OXM, and PYY seen post RYGB were successfully matched using 4 pmol/kg/min, 4 pmol/kg/min, and 0.4 pmol/kg/min, respectively. This dose led to a mean reduction of 32% in food intake. No significant effects on resting energy expenditure were observed.

**Conclusion:**

This is, to our knowledge, the first time that an acute continuous subcutaneous infusion of GOP, replicating the postprandial levels observed after RYGB, is shown to be safe and effective in reducing food intake. This data suggests that triple hormone therapy might be a useful tool against obesity.

## Teaser

A combined subcutaneous infusion of GLP-1, OXM, and PYY given to obese volunteers, matching the postprandial levels of the hormones as observed post RYGB, led to a 32% reduction in total food intake.

Obesity affects a quarter of the UK population, and at least a third of the world population is considered overweight or obese (1, 2). Bariatric surgery is currently the most effective treatment of obesity and related conditions such as type 2 diabetes (3, 4). Glucagon-like peptide-1 (GLP-1), Oxyntomodulin (OXM), and Peptide YY (PYY) are anorexia-inducing hormones secreted from the neuroendocrine L cells of the gut in response to nutrients (5–7). GLP-1 is also known to be insulinotropic, and OXM has the potential to increase energy expenditure (8). Postprandial secretion of the gut hormones GLP-1, OXM, and PYY is dramatically elevated after Roux-en-Y gastric bypass (RYGB) (7, 9, 10). It has been hypothesized that these hormonal changes importantly contribute to the significant and sustained weight loss observed post RYGB surgery (11, 12).

Intravenous infusion or subcutaneous injection of each of the individual three hormones at slightly higher concentrations has been shown to cause modest reductions in *ad libitum* food intake in humans (8, 13, 14). Studies involving intravenous coinfusion of PYY and GLP-1, or GLP-1 and OXM, demonstrated both additive and synergistic effects on food intake reduction (15–19). These studies suggest that both a single and dual combination of gut hormones are effective in reducing energy intake. In this study, a combination of GLP-1, OXM, and PYY (GOP) was administered to obese volunteers in a single-blinded, placebo-controlled study with a crossover design. The aim was to (1) replicate the observed postprandial levels of GLP-1, OXM, and PYY after RYGB, (2) assess the safety and tolerability of the GOP combination infusion, and (3) assess the effect of GOP on food intake and resting energy expenditure (REE). To achieve this in an ambulatory setting, GOP was infused continuously using a subcutaneous pump device. We conducted an initial dose-finding study in which we identified the doses of each peptide, which would simulate the postprandial RYGB milieu when infused in combination.

This study was also designed in anticipation of future studies in which volunteers might be wearing the pump in a free-living environment for 1 to 3 months. Continuous subcutaneous infusion of GLP-1 between 48 hours and 3 months has been described before. The majority of these studies demonstrated a significant improvement in glycemic parameters in overweight and obese volunteers with diabetes while demonstrating that the technique was safe and practical (20–23).

## Methods

### Participants

The study was designed as a randomized, single-blinded, placebo-controlled study with crossover arms. The study took place at the National Institute for Health Research/Wellcome Trust Clinical Research Unit Facility at Hammersmith Hospital. Ethical approval was obtained from the West London National Research Ethics Committee (reference number 13/LO/1510), and the study was conducted according to the principles of the Declaration of Helsinki. Obese healthy volunteers, between ages 18 and 65 years, with a body mass index (BMI) of over 30 kg/m^2^ were recruited for the infusion study. Volunteers were excluded if they were smokers (as this suppresses appetite) or possessed a history of a eating disorder as assessed by the SCOFF and Dutch Eating Behavior Questionnaires (24, 25).

### Study protocol

**Figure 1.**
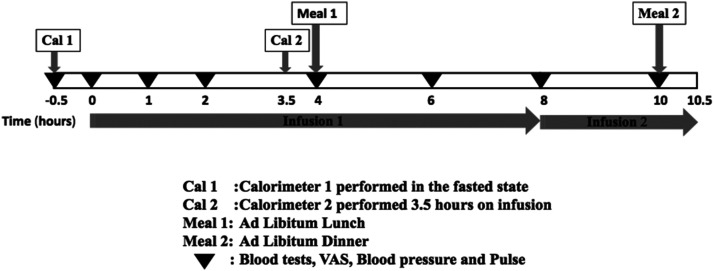
Study protocol.

Participants attended the research unit in the fasted state at 8 am on day 1 for a 3-day inpatient study visit. The study protocol is shown in Fig. 1. Day 1 consisted of a positive acclimatization visit whereby all volunteers received a lower dose GOP infusion (see below) to limit the effects of stress caused by subjects being unfamiliar with study procedures. A venous cannula was inserted in the antecubital fossa, and samples were taken for gut hormones, glucose, and insulin levels at the start of the subcutaneous pump infusion and subsequently at 1, 2, 4, 6, 8, and 10 hours. Visual analog scores (VASs) for sensations of “sickness,” “hunger,” “amount to eat,” and “fullness,” as well as pulse rate and blood pressure, were also recorded (26). A cannula attached to the infusion pump was positioned in the abdominal subcutis allowing for a 10.5-hour infusion. The pump was stopped after dinner. On day 2, volunteers were randomized in a single-blinded fashion to receive either GOP or 0.9% saline for 10.5 hours following the same protocol as on day 1. On day 3, the volunteers were given either GOP or 0.9% saline infusion, whichever had not been given on day 2.

### RYGB cohort

A separate surgical study cohort who had RYGB surgery at Imperial Weight Centre between 2007 and 2012 was also recruited for measurement of the reference gut hormone levels we would be targeting in our infusion study. The RYGB volunteers arrived at the unit at 9 am in the fasted state. A standardized mixed meal test (Ensure Plus; Abbott, Maidenhead, United Kingdom; 13.8 g of protein, 10.8 g of fat, 44.4 g of carbohydrates, 330 kcal, 220 mL) was given, and volunteers were directed to drink the Ensure Plus over 10 minutes. Blood was taken from an indwelling cannula placed in the antecubital fossa for gut hormone (GLP-1, OXM, and PYY), glucose, and insulin measurement at baseline and 30, 60, and 120 minutes post Ensure Plus ingestion.

### GOP infusion and doses

GLP-1 (7-36) amide, PYY (3-36), and OXM were purchased from Insight Biotechnology (London, United Kingdom). The freeze-dried peptides were reconstituted in saline and were shown to be stable for up to 12 hours at 37°C, as assessed by high-performance liquid chromatography. We changed the infusion to a freshly constituted solution after 8 hours on every study day to assure that the infused peptides were active. A portable Cane Crono infusion pump (Applied Medical Technology, Cambridge, United Kingdom) was used to deliver the peptides subcutaneously via an infusion set inserted under the anterior abdominal skin (Quick-set Infusion Set; Medtronic, Watford, United Kingdom). Gelofusine was used to prime the syringes and tubing prior to use to prevent adsorption of the peptides to tubing (27); however, the peptides were reconstituted in 0.9% saline for subcutaneous infusion. Following an initial dose-finding study, in which individual peptides were given for periods of up to 10 hours to volunteers to determine the steady-state plasma levels, we found that a combination of GLP-1 at 4 pmol/kg/min, OXM at 4 pmol/kg/min, and PYY at 0.4 pmol/kg/min was able to replicate the postprandial levels seen after RYGB. On day 2 or 3, these were given continuously for 10.5 hours. For the acclimatization visit on day 1, we started the infusion at a lower dose of 3 pmol/kg/min of both GLP-1 and OXM and 0.3 pmol/kg/min of PYY.

### *Ad libitum* food intake

*Ad libitum* food intake studies were performed at 4 and 10 hours during the infusion on each study day. A choice of homogenized meals of similar calorie density was served (Sainsbury’s Supermarkets Ltd, London, United Kingdom). Meal choices were served to volunteers at the screening visit, and volunteers were asked to rate their preference for each choice. Where volunteers recorded a dislike or extreme preference for a specific meal choice, these were excluded from their menu for the study days to minimize bias. Similar meals were served on study days 2 and 3 for direct comparison. Volunteers were asked to eat until comfortably full.

### Energy expenditure

Indirect calorimetry carried out with a ventilated hood system (Gas Exchange Monitor; GEM Nutrition, Daresbury, United Kingdom) was used to measure REE (19). Energy expenditure at baseline (prior to infusion start) and following 3.5 hours of GOP infusion or 0.9% saline (fasted state) was measured on each study day. Volunteers emptied their bladder prior to the baseline calorimeter, and urine was collected at the end of the second calorimeter for estimation of urinary nitrogen. Volunteers were asked to relax in the semisupine position for 30 minutes before each calorimeter recording. The rates of oxygen consumption and carbon dioxide production, $\overset{.}{V}$O_2_ and $\overset{.}{V}$CO_2_, respectively, were measured every minute, and estimation of energy expenditure was performed following the abbreviated Weir equation, with adjustment for urinary nitrogen excretion. Readings were taken for 15 to 20 minutes, allowing at least 10 minutes for the readings to stabilize. The mean of the five most stable, consecutive measurements was used in the analysis.

### Assays

Blood samples for gut hormone analysis were collected in lithium heparin collection tubes containing 0.1 mL of aprotinin for every 4 mL of blood. After centrifugation, plasma was stored at –80°C until analysis. Active GLP-1 and total PYY were measured by the MILLIPLEX magnetic bead–based multianalyte, metabolic panel, four-plex immunoassay (Millipore, St. Charles, MO), which was selective for the individual analyte. The intra-assay and interassay coefficient of variation for each analyte was <10%, and the lowest limit of detection for each peptide was 0.8 and 3.4 pmol/L for active GLP-1 and total PYY, respectively. OXM was measured using a specific and sensitive mass spectrometry validated assay (Holst Laboratory, University of Copenhagen, Copenhagen, Denmark) (7). Intra- and interassay precision (% CV) for the OXM assay was <10%.

### Statistical analysis

All analyses were carried out and figures produced using GraphPad Prism 6 (GraphPad Software, San Diego, CA). Descriptive data are expressed as the mean ± the standard error of the mean (SEM). A paired Student *t* test was used to compare food intake and energy expenditure between GOP and 0.9% saline infusion. Two-way repeated-measures analysis of variance (ANOVA) with a Bonferroni *post hoc* test was used to compare safety parameters and VASs across the two infusions, and one-way ANOVA with a Tukey *post hoc* test was used to compare gut hormone levels between the infusions and RYGB group.

The area under the curve (AUC) for glucose and insulin was calculated using the trapezoid rule, and comparison between GOP and 0.9% saline arms was made using the paired Student *t* test. The effect size was estimated using food intake data from pilot studies in our department. Using a significance level of 5% and a power of 80%, we estimated that 10 volunteers were sufficient to demonstrate a food reduction of 30% with GOP compared with 0.9% saline.

## Results

Ten volunteers were recruited for the infusion study (infusion group), and 8 volunteers who were 3 ± 0.6 years post RYGB were studied as the reference group (RYGB group). The two groups were matched for age and BMI (Table 1). None of the volunteers had diabetes at the time of the study, although one volunteer in the infusion group had impaired fasting glucose according to American Diabetes Association criteria. All volunteers in the surgical group achieved successful and stable weight loss of 32.2% ± 11.9% (mean ± standard deviation) at the time of the study, and four had diabetes prior to RYGB.

**Table 1.** Demographics of Infusion Group and RYGB Group

	**Infusion (n = 10)**	**RYGB (n = 8)**	***P* Value**
Female:male	7:3	5:3	
Age (years)	45.0 ± 11	46.6 ± 9.8	0.75
Weight (kg)	104.8 ± 22.9	88.8 ± 18.1	0.13
BMI (kg/m^2^)	36.3 ± 5.6	34.8 ± 4.9	0.65
HbA1c (%) (mmol/mol)	5.5 (36.3 ± 5.4)	5.5 (36.7 ± 3.1)	0.86

Data expressed as mean ± standard deviation.

Abbreviation: HbA1c, glycated hemoglobin.

### Food intake and VAS

**Figure 2.**
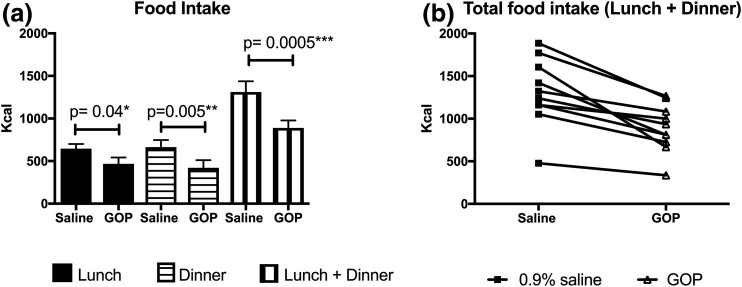
Food intake on GOP vs 0.9% saline (n = 10). (a) Food intake at lunch, dinner, and lunch plus dinner as measured on the GOP infusion day, compared with the 0.9% saline infusion day. Lunch and dinner served at *t* = 4 and *t* =10 hours, respectively. Mean ± SEM plotted. Paired Student *t* test shows significant differences between saline and GOP arms for lunch, dinner, and total (lunch plus dinner) food intake. (b) Individual total food intake on GOP vs 0.9% saline infusion.

**Figure 3.**
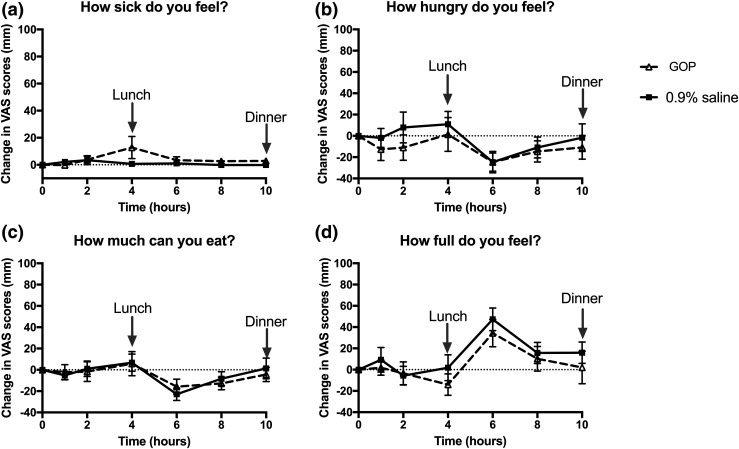
Change in VASs between GOP (dashed line, triangle symbols) and 0.9% saline (solid line, square symbols) infusion. Mean ± SEM plotted. (a) “How sick do you feel?” score. (b) “How hungry do you feel?” score. (c) “How much can you eat?” score. (d) “How full do you feel?” score. No significant differences observed between the two infusions on two-way repeated-measures ANOVA with Bonferroni *post hoc* test.

All volunteers ate less when GOP was infused (Fig. 2). Volunteers consumed 468.3 ± 73.8 kcal for lunch during GOP infusion vs 645 ± 55.9 kcal on 0.9% saline (*P* = 0.04). Food consumption for dinner was 420 ± 90.6 kcal during GOP infusion vs 664.7 ± 83.5 kcal on 0.9% saline (*P* = 0.005). Total food intake over 10 hours of infusion was thus significantly lower on GOP compared with 0.9% saline (888.9 ± 89.3 kcal vs 1310 ± 127 kcal, respectively; *P* = 0.0005). This represents a mean reduction of total food intake of 32% or 421.1 kcal. There was no significant difference in the food intake reduction when the volunteers who had 0.9% saline first were compared with those who had GOP infusion first (Supplemental Table 1). Three volunteers experienced mild nausea with GOP, which eventually settled without requiring any intervention within the first 2 to 4 hours of the infusion, and none with 0.9% saline infusion. However, there was statistically no significant difference between nausea scores as recorded on VASs between the two infusions. Sensations of “hunger,” “amount to eat,” and “fullness” scores were also not significantly different (Fig. 3).

### GOP blood levels

**Figure 4.**
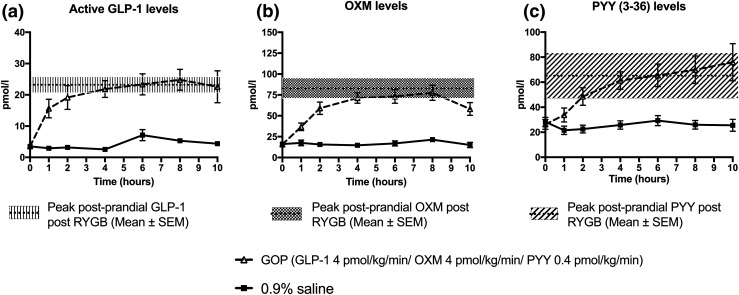
GLP-1, OXM, and PYY levels achieved following GOP and 0.9% saline infusion. (a) GLP-1 levels. Peak postprandial levels of GLP-1 in RYGB group matched by GOP infusion. (b) OXM levels. Peak postprandial levels of OXM in RYGB matched by GOP infusion. (c) Total PYY levels. Peak postprandial levels of PYY in RYGB matched by GOP infusion.

The circulating levels of GOP achieved on 4 pmol/kg/min of GLP-1, 4 pmol/kg/min of OXM, and 0.4 pmol/kg/min of PYY infused subcutaneously over 10.5 hours are shown in Fig. 4. Baseline levels of GLP-1 were comparable between all three groups (GOP arm: 3.5 ± 0.6 pmol/L; RYGB group: 4.3 ± 1.2 pmol/L; 0.9% saline arm: 3.5 ± 0.6 pmol/L; *P* = 0.78 for comparison between GOP arm and RYGB group and *P* = 1.0 for comparison between GOP arm and 0.9% saline arm) and similarly for baseline PYY (GOP arm: 26.3 ± 4.0 pmol/L; RYGB group: 21.6 ± 4.2 pmol/L; 0.9% saline: 28.3 ± 3.6 pmol/L; *P* = 0.70 for comparison between GOP arm and RYGB group and *P* = 0.93 for comparison between GOP arm and 0.9% saline arm). Baseline OXM levels were nonsignificantly lower in the RYGB group compared with GOP (RYGB group: 10.5 ± 1.7 pmol/L vs GOP arm: 15.8 ± 2.3 pmol/L; *P* = 0.2) and comparable between the GOP and 0.9% saline arms (0.9% saline arm: 16.2 ± 2.3 pmol/L; *P* = 0.99). As expected, the RYGB group showed at least a fourfold increase in postprandial levels of each peptide following a standardized mixed meal. The peak postprandial levels of each hormone in the RYGB group closely matched the peak levels achieved by GOP infusion (RYGB vs GOP, respectively: GLP-1: 26.3 ± 3.5 pmol/L vs 26.1 ± 3.2 pmol/L, *P* = 1.0; OXM: 94.3 ± 10.5 pmol/L vs 83.8 ± 6.8 pmol/L, *P* = 0.56; PYY: 81.3 ± 17.8 pmol/L vs 79.2 ± 11.2 pmol/L, *P* = 0.99). These levels were achieved within 4 hours of starting the infusion. As expected, peak GLP-1, OXM, and PYY levels in the 0.9% saline infusion arm were significantly lower compared with the RYGB and GOP groups (0.9% saline GLP-1: 7.0 ± 1.4 pmol/L, *P* < 0.0001 compared with GOP and *P* < 0.001 compared with RYGB; 0.9% saline OXM: 24.6 ± 3.0 pmol/L, *P* < 0.0001 compared with GOP and *P* < 0.0001 compared with RYGB; 0.9% saline PYY: 28.9 ± 3.6 pmol/L, *P* < 0.01 compared with GOP and *P* < 0.05 compared with RYGB).

### Energy expenditure

REE was measured in seven volunteers. The change in REE between baseline and infusion phases was not significantly different between GOP and 0.9% saline infusions (–4.6 ± 74.2 kcal/24 h vs 22.0 ± 68.8 kcal/24 h, *P* = 0.78).

### Safety parameters

**Figure 5.**
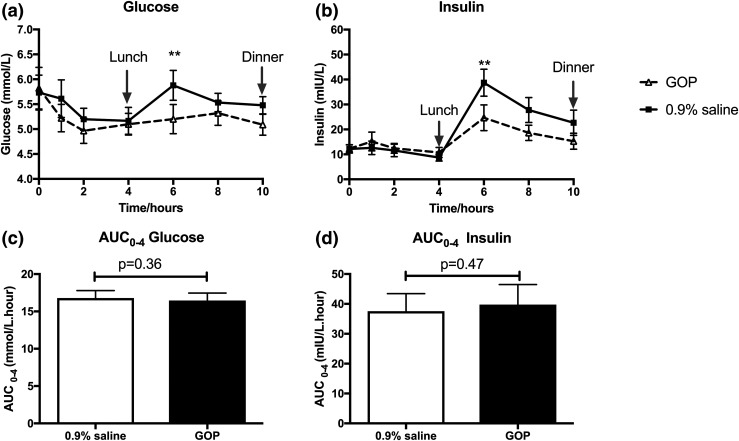
The effects of GOP (dashed line, triangle symbols) and 0.9% saline infusion (solid line, square symbols) on glucose and insulin profile. (a) Glucose levels at 6 hours significantly higher on 0.9% saline infusion. (b) Insulin levels at 6 hours significantly higher on 0.9% saline infusion. Mean ± SEM plotted with two-way repeated-measures ANOVA with Bonferroni *post hoc* test: ***P < 0.001. (c and d) No significant difference observed between glucose and insulin AUCs over the first 4 hours. Paired Student *t* test applied to compare AUCs calculated for glucose and insulin over the first 4 hours of infusion (fasted state).

We observed no significant difference in systolic and diastolic blood pressure profiles as well as pulse rate between GOP and 0.9% saline infusion (Supplemental Fig. 1). Mean fasting glucose was 5.9 ± 0.4 mmol/L and 5.7 ± 0.3 mmol/L and mean fasting insulin was 13.4 ± 1.9 mIU/L and 13.9 ± 1.3 mIU/L on GOP and 0.9% saline infusion days, respectively. During the first 4 hours of the infusion (fasted state), glucose levels were comparable between both arms. However, by 6 hours (2 hours after lunch), glucose levels diverged and were significantly higher in the 0.9% saline arm (5.2 ± 0.3 mmol/L vs 6.0 ± 0.3 mmol/L, *P* < 0.001). Insulin levels also followed a similar pattern. Fasting insulin levels were comparable during the first 4 hours of the infusion in both arms, but by 6 hours, insulin levels were significantly higher in the 0.9% saline arm (24.3 ± 4.6 mIU/L vs 43.3 ± 6.7 mIU/L, *P* < 0.001; Fig. 5). Importantly, there was no episode of hypoglycemia in either infusion arm.

## Discussion

We have demonstrated that a continuous subcutaneous infusion of the peptides GLP-1, OXM, and PYY can be safely and successfully given to obese, nondiabetic volunteers in an ambulatory setting. Using doses of 4 pmol/kg/min of GLP-1, 4 pmol/kg/min of OXM, and 0.4 pmol/kg/min of PYY, we were able to reach target levels of each hormone comparable to the postprandial levels observed in our BMI-matched RYGB cohort. As expected, with subcutaneous delivery, it took longer to achieve stable levels in comparison with intravenous infusions in previous studies (13, 28–30), but these levels were stably maintained for several hours in ambulatory patients. Previous studies have infused subcutaneous GLP-1 at doses between 1.2 pmol/kg/min and 8.5 pmol/kg/min (21, 22). Toft-Nielsen *et al.* achieved mean active GLP-1 levels of 22.5 ± 3.7 pmol/L following a dose of 2.4 pmol/kg/min, which is comparable to what we measured on 4 pmol/kg/min (26.1 ± 3.2 pmol/L) (20). This disparity in doses is likely to be due to differences in study protocol and methods: They infused only GLP-1 continuously for a longer period of 48 hours, and there were differences in the assays used.

Energy intake was reduced by 32% during 10.5 hours of GOP infusion compared with 0.9% saline. During our initial dose finding with infusions of individual peptides, we found that subcutaneous infusion doses of 12 pmol/kg/min for GLP-1, 30 pmol/kg/min for OXM, and 1 pmol/kg/min for PYY, respectively, were necessary to achieve comparable food intake reductions of 21% to 37% (Supplemental Fig. 2). In other words, when combined, the three peptides achieved a profound food intake reduction with much reduced doses. The food intake reduction observed in the current study is also in keeping with other combination intravenous infusion studies; for example, De Silva *et al.* and Schmidt *et al.* both found a reduction of 27% and 30% in food intake, respectively, when GLP-1 and PYY were infused in combination (15, 16), and Field *et al.* infused OXM and GLP-1 in combination, which resulted in a similar reduction in food intake (18). The key differences between the current study and these previous studies are as follows: (1) This is, to our knowledge, the first time that such effects have been demonstrated with continuous subcutaneous infusion of a triple combination of GLP-1, OXM, and PYY at circulating levels comparable to those observed postprandially in RYGB patients, as opposed to the more elevated levels seen in previous infusion studies; (2) this study used an infusion over an extended period of time (10.5 hours) compared with previous studies employing shorter durations of 120 to 150 minutes (15, 16), indicating that this can potentially be given chronically for a sustained effect on energy intake and subsequent weight loss; and (3) the volunteers were fully ambulatory during the infusion, which is more in keeping with a “free-living” setting.

GLP-1 and PYY are known to have powerful anorexic effects by binding to GLP-1 and Neuropeptide Y2 receptors, respectively, located mainly within the hypothalamus in studies in rodents and humans (5, 6, 31). However, OXM is believed to exert its anorexic effects via the GLP-1 and Glucagon receptors, albeit it has a much lower binding affinity for these receptors compared with their cognate peptides. GLP-1 and PYY are likely to be the main contributors toward the reduction in food intake observed in our study. Our current study was not designed to disentangle the contribution of each peptide toward the reduction in energy intake observed nor to assess any additive or synergistic effects of a triple-hormone infusion but rather to investigate the effects of a postprandial RYGB hormonal milieu on energy intake in a nonsurgical cohort. Nevertheless, the reduction in energy intake observed in our study, if sustained long term, could contribute to greater magnitudes of weight loss compared to current therapies where weight loss of up to 10% is typically achieved (32–35).

We did not find any statistically significant change in REE following GOP infusion at the doses used in this study, consistent with the study of Bagger *et al.*, who showed that IV infusions of GLP-1 and OXM do not change REE as assessed by calorimetry (36). Limitations with respect to this observation include the fact that our study was not specifically powered for this outcome and the study design did not allow for measurement of REE beyond 4 hours of the infusion, as volunteers were then fed. Although OXM has been shown to induce an increase in activity-related energy expenditure following subcutaneous injection (8), the protocol did not measure this specific aspect of energy expenditure.

Three out of 10 volunteers experienced mild nausea during the first 2 to 4 hours of the GOP infusion. The nausea spontaneously resolved, and volunteers remained free of nausea for the remaining part of the infusion. The nausea observed in our study is unlikely to have influenced food intake, as has previously been found with GLP-1 analogs, and statistically, there was no significant difference between the VASs for “sickness” on GOP compared with 0.9% saline. In addition, none of the volunteers experienced any nausea during the latter 6 hours of the infusion, and food intake at both lunch and dinner were reduced equally by about a third. Volunteers receiving GOP tended to be less “hungry” during the first 4 hours of the infusion, although this did not reach statistical significance.

There was no safety concern in relation to hemodynamic parameters during GOP infusion. Subcutaneous GLP-1, at 2.4 pmol/kg/min, has previously been shown to slightly lower blood pressure possibly through relaxation of the vasculature and through diuresis (20). However, we did not observe a significant change in blood pressure.

We did not observe any significant reductions in glucose or insulin secretion enhancement with GOP compared with the 0.9% saline arm. This may be explained by the fact that our volunteers did not have diabetes, and that GLP-1’s insulinotropic effects are glucose level dependent. In the postprandial state, volunteers on 0.9% saline displayed a significantly higher glucose and insulin response following lunch at 6 hours; this is likely to be related to increased consumption of the test meal compared with the GOP arm. Delayed gastric emptying in volunteers receiving GOP may also be a contributing factor to these observed differences between the two groups. In contrast, significant improvement in glycemia, including insulin sensitivity, was observed during GLP-1 subcutaneous infusion in patients with type 2 diabetes (21, 22).

Some limitations to this study should be noted, including a relatively small sample size (which was powered to detect a significant difference in food intake) and the single-blinded design. The continuous infusion of GOP does not reflect the patterns of peaks and troughs of gut hormones seen after RYGB. The postprandial elevation in gut hormones usually peaks around 30 minutes after ingestion with levels returning to baseline by 3 hours (11). We chose not to give a bolus of GOP with meals because a rapid rise in circulating levels is more likely to induce nausea. Furthermore, RYGB patients tend to have smaller but more frequent meals (five to six a day), thus experiencing regular elevations in gut hormone secretion (37). This leads to a very similar overall exposure to elevated levels of gut hormones between RYGB and our current GOP infusion.

In conclusion, our findings suggest that the postprandial elevations in GLP-1, OXM, and PYY observed after RYGB can be recapitulated with a subcutaneous triple-hormone infusion, leading to a profound reduction of food intake. We also speculate that this could be exploited in future treatments for obesity and diabetes.
