# Supplementary material for: The Effect of a Subcutaneous Infusion of GLP-1, OXM, and PYY on Energy Intake and Expenditure in Obese Volunteers
Source: J Clin Endocrinol Metab. 2017 Apr 4;102(7):2364–72. doi: 10.1210/jc.2017-00469 (PMC5505203; doi:10.1210/jc.2017-00469)
Supplement: Supplementary file 1 [file jc.2017-00469.st1.docx]

**Supplemental Table 1: Food intake.** Change in food intake for GOP preceded by 0.9% saline (-439.8 ± 95 kcal) was not significantly different to 0.9% saline preceded by GOP (-402.6 ± 138 kcal), p=0.83 (Mean ± SEM and Unpaired Student t-test applied).

| Order of infusion | Volunteer | Food intake (kcal) | | |
| --- | --- | --- | --- | --- |
|  |  | 0.9% saline infusion | GOP infusion | Change (GOP-0.9% saline) |
| GOP preceded by 0.9% saline | 1 | 1773 | 1268 | -505 |
|  | 5 | 1885 | 1246 | -639 |
|  | 6 | 1421 | 811 | -610 |
|  | 7 | 478 | 336 | -142 |
|  | 9 | 1239 | 936 | -303 |
| 0.9% saline preceded by GOP | 2 | 1605 | 667 | -938 |
|  | 3 | 1053 | 726 | -327 |
|  | 4 | 1323 | 1085 | -239 |
|  | 8 | 1160 | 1000 | -160 |
|  | 10 | 1162 | 813 | -349 |
