# Supplementary figures and images for: The Effect of a Subcutaneous Infusion of GLP-1, OXM, and PYY on Energy Intake and Expenditure in Obese Volunteers
Source: J Clin Endocrinol Metab. 2017 Apr 4;102(7):2364–72. doi: 10.1210/jc.2017-00469 (PMC5505203; doi:10.1210/jc.2017-00469)

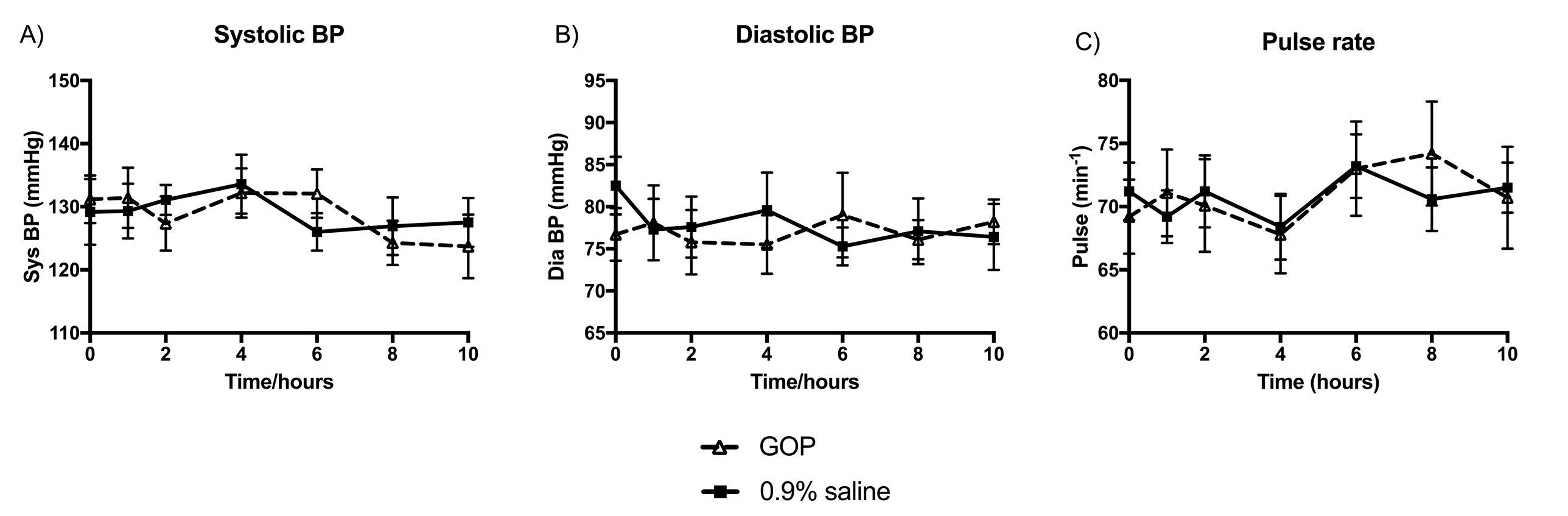

Supplement: Supplementary file 2 [file jc.2017-00469.sf1.tiff]
